# *Rhodnius prolixus* interaction with *Trypanosoma rangeli*: modulation of the immune system and microbiota population

**DOI:** 10.1186/s13071-015-0736-2

**Published:** 2015-03-01

**Authors:** Cecilia S Vieira, Débora P Mattos, Peter J Waniek, Jayme M Santangelo, Marcela B Figueiredo, Marcia Gumiel, Fabio F da Mota, Daniele P Castro, Eloi S Garcia, Patrícia Azambuja

**Affiliations:** Laboratório de Bioquímica e Fisiologia de Insetos, Instituto Oswaldo Cruz, Fundação Oswaldo Cruz (IOC/FIOCRUZ), Rio de Janeiro, RJ Brazil; Departamento de Entomologia Molecular, Instituto Nacional de Entomologia Molecular (INCT-EM), Rio de Janeiro, RJ Brazil; Departamento de Ciências Ambientais, Instituto de Florestas, Universidade Federal Rural do Rio de Janeiro (UFRRJ), Seropédica, RJ Brazil

**Keywords:** *Rhodnius prolixus*, *Trypanosoma rangeli*, Immune system, Prophenoloxidase, Antimicrobial peptide

## Abstract

**Background:**

*Trypanosoma rangeli* is a protozoan that infects a variety of mammalian hosts, including humans. Its main insect vector is *Rhodnius prolixus* and is found in several Latin American countries. The *R. prolixus* vector competence depends on the *T. rangeli* strain and the molecular interactions, as well as the insect’s immune responses in the gut and haemocoel. This work focuses on the modulation of the humoral immune responses of the midgut of *R. prolixus* infected with *T. rangeli* Macias strain, considering the influence of the parasite on the intestinal microbiota.

**Methods:**

The population density of *T. rangeli* Macias strain was analysed in different *R. prolixus* midgut compartments in long and short-term experiments. Cultivable and non-cultivable midgut bacteria were investigated by colony forming unit (CFU) assays and by 454 pyrosequencing of the 16S rRNA gene, respectively. The modulation of *R. prolixus* immune responses was studied by analysis of the antimicrobial activity *in vitro* against different bacteria using turbidimetric tests, the abundance of mRNAs encoding antimicrobial peptides (AMPs) defensin (*DefA*, *DefB*, *DefC*), prolixicin (*Prol*) and lysozymes (*LysA*, *LysB*) by RT-PCR and analysis of the phenoloxidase (PO) activity.

**Results:**

Our results showed that *T. rangeli* successfully colonized *R. prolixus* midgut altering the microbiota population and the immune responses as follows: 1 - reduced cultivable midgut bacteria; 2 - decreased the number of sequences of the Enterococcaceae but increased those of the Burkholderiaceae family; the families Nocardiaceae, Enterobacteriaceae and Mycobacteriaceae encountered in control and infected insects remained the same; 3 - enhanced midgut antibacterial activities against *Serratia marcescens* and *Staphylococcus aureus;* 4 - down-regulated *LysB* and *Prol* mRNA levels; altered *DefB*, *DefC* and *LysA* depending on the infection (short and long-term); 5 - decreased PO activity.

**Conclusion:**

Our findings suggest that *T. rangeli* Macias strain modulates *R. prolixus* immune system and modifies the natural microbiota composition.

## Background

The haemoflagellate, *Trypanosoma rangeli,* is a protozoan parasite that infects a large number of mammals, including humans, and it is vectored by triatomine insects, especially the genus *Rhodnius* [1-4]. The interaction with triatomine hosts, such as *Rhodnius prolixus,* begins with the ingestion of an infective blood meal containing *T. rangeli.* After ingestion, the parasites transform into epimastigotes, then multiplies in the insect gut, and invades the haemolymph. To perpetuate the infection they transform into the metacyclic forms in the salivary glands [1,2,5]. The life cycle of *T. rangeli* is completed with the transmission of the parasite to vertebrate hosts by the vector through its salivary gland secretions during a blood meal [6,7].

The establishment of *T. rangeli* infections in both the digestive tract and haemocoel is regulated by physiological processes of the triatomine vector [8]. The parasites survive despite the activation of innate immune reactions and complete their life cycle in the insect host [9-11]. Once inside the midgut, the parasites must interact with blood digestion products as well as midgut components including bacterial microbiota [12,13], haemolytic factors [14,15], lectins [16], the prophenoloxidase (PPO) system [17], antimicrobial peptides (AMPs) [18,19] and reactive nitrogen and oxygen species [20].

Some of these factors act as biological barriers to the infection of *T. rangeli* in the vector gut. However, the *T. rangeli* infection may lead to immunedepression of the insect host by the inhibition of phagocytosis, haemocyte microaggregation, PPO activation and eicosanoids synthesis [11,21,22]. These physiological alterations allow the parasites to overcome the immune response, reach the salivary glands and complete their life cycle.

Knowledge of the modulation of the triatomine immune system by the numerous strains of *T. rangeli* is still poorly understood. Thus, the aim of the present study was to investigate the effects of *T. rangeli* Macias strain infection on the midgut immune responses, parasite development and bacteria population of 5^th^ instar nymphs of *R. prolixus* orally infected with parasites. In addition to the evaluation of the effects of *T. rangeli* in short-term infections, long-term infections were analysed in the 5^th^ instar nymphs previously infected in the 4^th^ instar stage. The present results suggest that the parasites modulate the *R. prolixus* immune responses, affecting the intestinal microbiota by inhibiting activation of prophenoloxidase, altering the abundance of antimicrobial peptide transcripts and enhancing antimicrobial activities against *Serratia marcescens*. These results provide further elucidation of the *T. rangeli*-*R. prolixus* interaction.

## Methods

### Ethics Statement

Defibrinated rabbit blood provided by the Animals Creation Center Laboratory (Cecal/Fiocruz) was provided to the insects through an artificial apparatus respecting the guidelines of the Ethics Committee on Animal Use (Ceua/Fiocruz). CEUA follows the Ethical Principles in Animal Experimentation composed by Fiocruz researchers and external consultants. The protocol number L-0061/08 was established by CONCEA/MCT [23].

### Maintenance of *Trypanosoma rangeli* epimastigotes

*T. rangeli* Macias strain, first isolated from a human in Venezuela [24,25] and later characterized as genotype KP1+ [26], was kindly supplied by Dr. Suzete Gomes, Universidade Federal Fluminense (Rio de Janeiro, Brazil). The parasites were maintained at 28°C in brain heart infusion (BHI) media (Sigma-Aldrich, São Paulo, Brazil) supplemented with 20% heat-inactivated bovine foetal serum [27] and subcultured twice a week. This procedure keeps the parasite in the log phase growth resulting predominantly in short epimastigotes (99%). The number of parasites was quantified in a Neubauer chamber.

### Bacteria preparation

*Staphylococcus aureus* 9518, and *Escherichia coli* K12 4401 were purchased from the National Collection of Industrial and Marine Bacteria (NCIMB), Aberdeen, UK. *S. marcescens* RPH was previously isolated from *R. prolixus* [12] and maintained at Laboratório de Bioquímica e Fisiologia de Insetos. The bacteria were maintained at -70°C in tryptone agar and 10% glycerol. For all experimental procedures, bacteria were grown as previously described [28]. Briefly, bacteria were grown overnight in tryptone soy broth (TSB) at 30°C and then cultured in fresh TSB for a further 4 h under the same conditions. The bacteria were then washed in phosphate buffered saline (PBS, 0.01 M phosphate buffer, 2.7 mM potassium chloride and 0.137 M sodium chloride, pH 7.4) and resuspended in TSB to a final concentration of 1 × 10^4^ cells/ml.

### Insect oral infection: long and short-term infections

Insects were kept at 27°C and fed artificially with defibrinated rabbit blood [27]. All insects were fed on blood, after heat-inactivation of the plasma. The blood was centrifuged at 1,890 x g for 10 min at 4°C and the supernatant (plasma) was incubated for 30 min at 55°C. The plasma was added back to the erythrocytes and then received *T. rangeli* epimastigotes obtained from culture. The same procedure of plasma inactivation was undertaken for control insects.

For long-term experiments, inactivated blood containing 1 × 10^6^ epimastigotes/mL (infected group) or blood without parasites (control uninfected group) was given to 4^th^ instar nymphs. After moulting to 5^th^ instars, both insect groups received a non-infective blood meal which occurred 38 days after feeding (DAF) of the 4th instar nymphs. For short-term experiments, 5^th^ instar nymphs were fed on blood containing 1×10^6^ epimastigotes/mL or with parasite-free blood. Only fully engorged insects were used for all experiments.

### Quantification of parasites in the digestive tract

Fifth instar nymphs obtained from long or short-term experiments were dissected. The anterior midgut (stomach) was collected and homogenized in 1.0 mL PBS and the posterior midgut (intestine) plus rectum was placed in 50 μL in PBS. Samples were macerated and the number of parasites was determined by counting in a Neubauer haemocytometer and expressed as parasites/mL. Parasites were quantified in three experiments with five insects each (n = 15).

### Analysis of intestinal microbiota

#### Colony forming unit (CFU)

Anterior midgut contents obtained from 5^th^ instar nymphs infected or uninfected with parasites (short and long-term infections) were analysed for microbiota bacterial population using CFU at 12 DAF. The midgut contents were serially tenfold diluted with PBS and 20 μL was spread on a Petri dish in BHI agar (Sigma-Aldrich) culture medium. The plates were incubated at 30°C for 24 h and the CFU counted. As a control, PBS was also plated to check the sterility of all experiments.

#### Metagenomic DNA extraction

Seven days after insect feeding (long-term infection), metagenomic DNA was extracted from the anterior midgut contents of four *T. rangeli* infected insects and four uninfected *R. prolixus* 5^th^ instar nymphs by an unbiased and efficient mechanical lysis method [29]. The extraction was carried out using the commercial Fast-DNA™ Spin Kit for Soil (Qbiogene, MP Biomedicals, USA) following the manufacturer's instructions. DNA extracts were visualized on 1% agarose gels to assess their integrity and purity.

#### Amplification and 454 sequencing of targeted 16S rRNA gene variable region

For quantitative analysis of bacterial microbiota in long-term infected insects, ribosomal genes from metagenomic DNA samples were amplified using bar-coded primers for the 16S variable region V3-V1, cleaned up, quantified and normalized according to the HMP 3730 16S protocol version 4.2 [30], which is available on the HMP Data Analysis and Coordination Center website [31]. The PCR products obtained were then submitted to FLX-Titanium pyrosequencing in a GS Junior System (Roche).

The raw sequences were analysed using the RDP Pipeline with default parameters. Sequences with a score below the quality threshold were discarded and the sequence portions devoted to 454 sequencing were trimmed out. Sequences with more than 400 bases were then aligned using the INFERNAL aligner [32] and chimeric sequences detected (and removed) with UCHIME [33]. Taxonomical classification was assigned using the RDP classifier [34,35] with a minimum confidence level for record assignment set to 0.80.

### Turbidimetric antibacterial assay

In long and short-term experiments, the antibacterial activities of the anterior midgut contents from 5^th^ instar nymphs infected or not with *T. rangeli* were tested at 7 DAF, according to previous studies which have shown that the maximal antibacterial activity is reached at this time [17,36]. Fifth instar nymphs of *R. prolixus* were dissected to collect the anterior midgut. The midgut walls were removed and the midgut contents pooled (3 insects) in 200 μl ultrapure water, homogenized, centrifuged at 10,000 x g for 10 min at 4°C and filtered by a sterile PVDF membrane (Millipore) and stored at −20°C until use. Before assaying, the midgut content samples were diluted ten times in sterile water. Subsequently, 10 μl of *E. coli*, *S. aureus or S. marcescens* bacterial suspensions (10^4^ cells/mL) were added to each well of a sterile flat bottom 96-well microtiter plate (Nunc, Fisher Scientific, Leicestershire, UK) with 45 μl of diluted midgut samples and 5 μl of peptone 10% and incubated at 37°C for 19 h. The optical densities were measured at 550 nm (OD_550_) at hourly intervals using a Spectra Max 190 Plate Reader (Molecular Devices, Sunnyvale, USA). Control wells, run without anterior midgut samples, contained 10 μl of bacteria in a final concentration of 1% peptone in ultrapure water. Ampicillin (80 μg/ml) was included in each experiment as an antibiotic control. To exclude the opacity of the midgut samples all data points were blanked against time zero. The antibacterial activity was calculated by subtracting the bacterial growth readings (control wells) from the respective values of anterior midgut samples incubated with bacteria.

#### Transcript abundance of antimicrobial peptides

Transcript abundance of genes encoding antibacterial peptides in short and long-term experiments with *T. rangeli* infected and uninfected 5^th^ instar nymphs was analysed by reverse transcription PCR (RT-PCR) as described previously [36]. In brief, the anterior and posterior midgut walls of a pool of ten insects were dissected from 5^th^ instar nymphs 1 and 7 DAF. Total RNA was extracted using the NucleoSpin® RNA II Kit (Macherey-Nagel, Düren, Germany), following the manufacturer’s instructions. RNA concentration was measured on a NanoDrop 2000 (Thermo Scientific, Waltham, USA). For cDNA synthesis, 1.25 or 2.5 μg of total RNA was performed using a First-Strand cDNA Synthesis Kit (GE Healthcare, Buckinghamshire, UK). Oligonucleotide primers for amplification of defensin A, B and C, lysozyme A and B, prolixicin [19,37,38] and ß-actin (endogenous control) were used. PCRs were carried out in triplicate on a Veriti 96 thermocycler (Applied Biosystems, Carlsbad, USA) using an IllustraTaq DNA-Polymerase (GE Healthcare). In negative PCR controls, ultrapure water was added instead of cDNA. PCR products were electrophoretically separated on a 2% agarose gel and stained with ethidium bromide. Gels were documented using an E-Gel® Imager (Life Technologies, Carlsbad, USA) and band intensity quantified using the ImageJ program (v. 1.47q).

#### Determination of phenoloxidase activity

Phenoloxidase (PO) activities were analysed in samples of the anterior midgut contents freshly prepared from 5^th^ instar nymphs obtained from long and short-term experiments. Each midgut content was diluted in 200 μL of ultrapure water, centrifuged at 10,000 x g for 10 min and the supernatant ten times diluted. For PO analysis, five insects were used from each group. The experiments were carried out in triplicate and at 7 and 12 DAF.

PO activity was determined by measuring the dopachrome formation from DOPA using midgut samples, as described previously [39]. For assaying, 25 μL of a midgut preparation was mixed with 10 μL of cacodylate-CaCl_2_ buffer (10 mM sodium cacodylate, 10 mM CaCl_2_, pH 7.4). After the addition of 25 μL of a saturated solution of DOPA (4 mg/mL), the absorbance at 490 nm was measured continually in a Spectra Max 190 Microplate Reader at 37°C for 120 min. The enzyme unit was expressed as absorbance/min × 100.

#### Statistical analysis

Depending on the distribution of the data and treatment number, the results obtained were analysed using 1-way ANOVA, Student’s *T*-test, the Kruskal-Wallis test or the Mann–Whitney test on GraphPad Prism 5 software. Differences between groups were considered statistically significant when p < 0.05. The levels of probability are shown in the respective figures.

## Results

### Short-term infection

#### Quantification of parasites in the digestive tract

**Figure 1:**
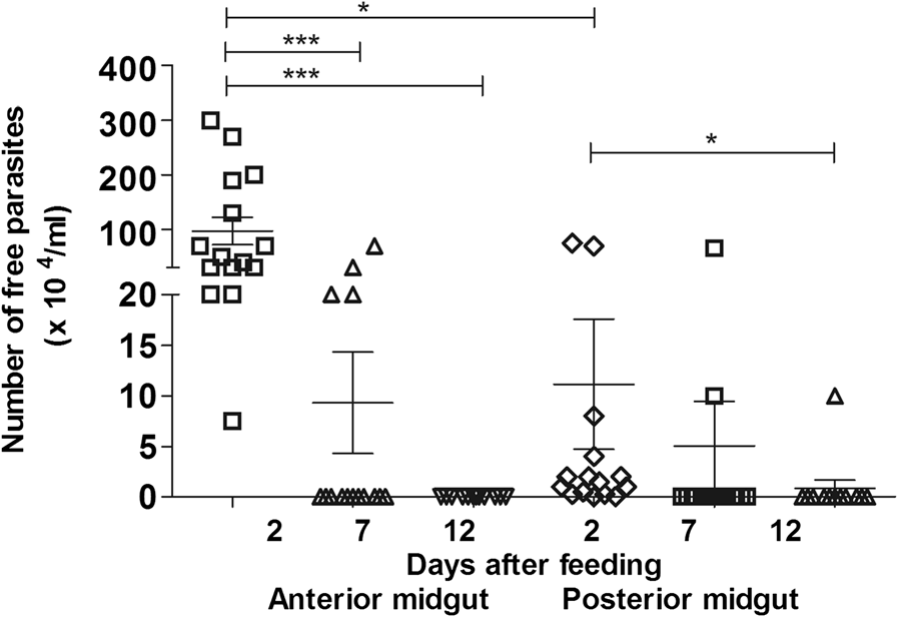
**Short-term experiment showing** ***Trypanosoma rangeli*** **numbers in the gut of** ***Rhodnius prolixus.*** Parasite numbers in *R. prolixus* 5^th^ instar nymphs were analysed in anterior midgut and posterior midgut plus rectum at different days after feeding. The insects were fed on inactivated blood containing 1 × 10^6^ epimastigotes/mL  *T. rangeli* Macias strain. Bars represent mean ± SEM of three independent experiments each with five insects (n=15). Means were compared using one-way ANOVA and Kruskal-Wallis test; ***p < 0.001 and *p < 0.05.

The infection rates of the *R. prolixus* digestive tract by *T. rangeli* were analysed on different days after feeding (DAF). Analyses of the presence of parasites in the digestive tract showed that the percentage of infected insects at 2 DAF was 100% and decreased to 26.6% and 6.7% after 7 and 12 days, respectively. The anterior midgut presented a high number of parasites, starting with 9.7 × 10^5^/mL at 2 DAF and decreased significantly along time to 9.3 × 10^4^/mL and 0, respectively, at 7 and 12 DAF (p < 0.001) (Figure 1). A similar pattern of parasite temporal distribution was observed in the posterior midgut and rectum with an infection level of 1.1 × 10^5^/mL, 5.0 × 10^4^/mL and 8.3 × 10^3^/mL at 2, 7 and 12 DAF, respectively with significant difference between 2 and 12 DAF (p < 0.05) (Figure 1).

#### Analysis of intestinal microbiota (CFU)

**Figure 2:**
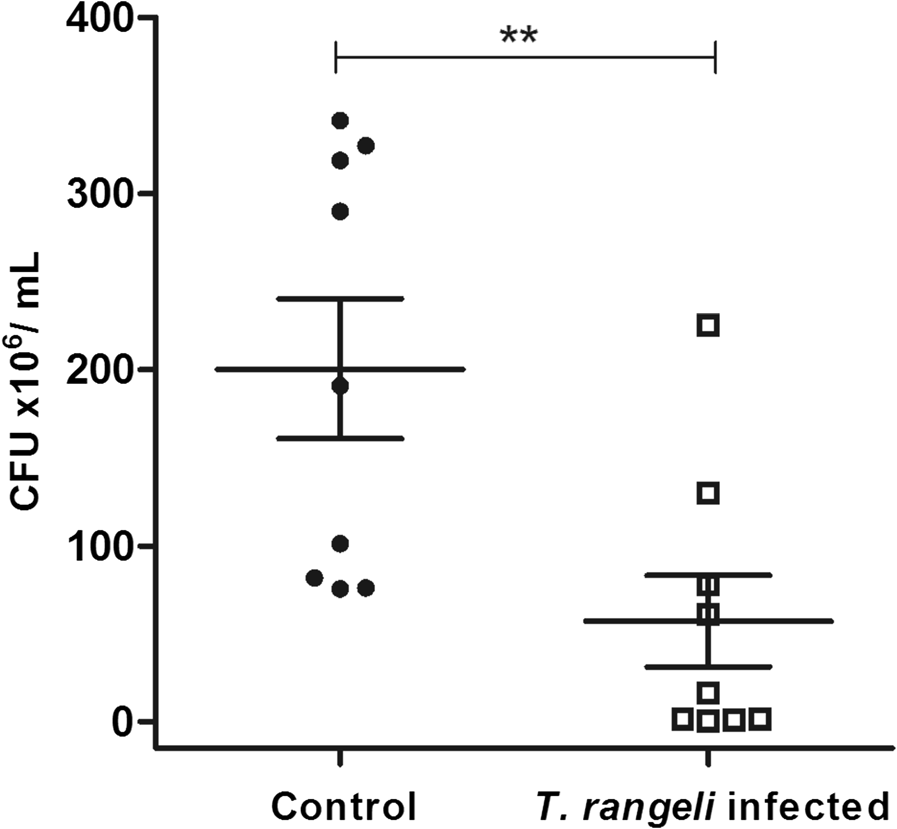
**Short-term experiment showing bacteria population in** ***Rhodnius prolixus*** **midgut infected with** ***Trypanosoma rangeli*** **.** Fifth instar nymphs were fed on inactivated blood containing 1 × 10^6^ epimastigotes/mL of *T. rangeli*. CFU counts were made 12 days after feeding. Bars represent mean ± SEM of three independent experiments with three pools of insects (n = 9). Each point represents the CFU from one pool of three insects. Means were compared using Student *T*-test or Mann–Whitney test; **p < 0.01.

Cultivable bacterial microbiota population in 5^th^ instar nymphs infected with *T. rangeli* Macias strain was evaluated using CFU counts of digestive tract preparations. At 12 DAF the bacterial population in infected insects (5.7 × 10^7^ CFU/mL) was significantly lower than the uninfected control (2.0 × 10^8^ CFU/mL) (p < 0.01) (Figure 2).

#### Turbidimetric (TB) antibacterial assay

**Figure 3:**
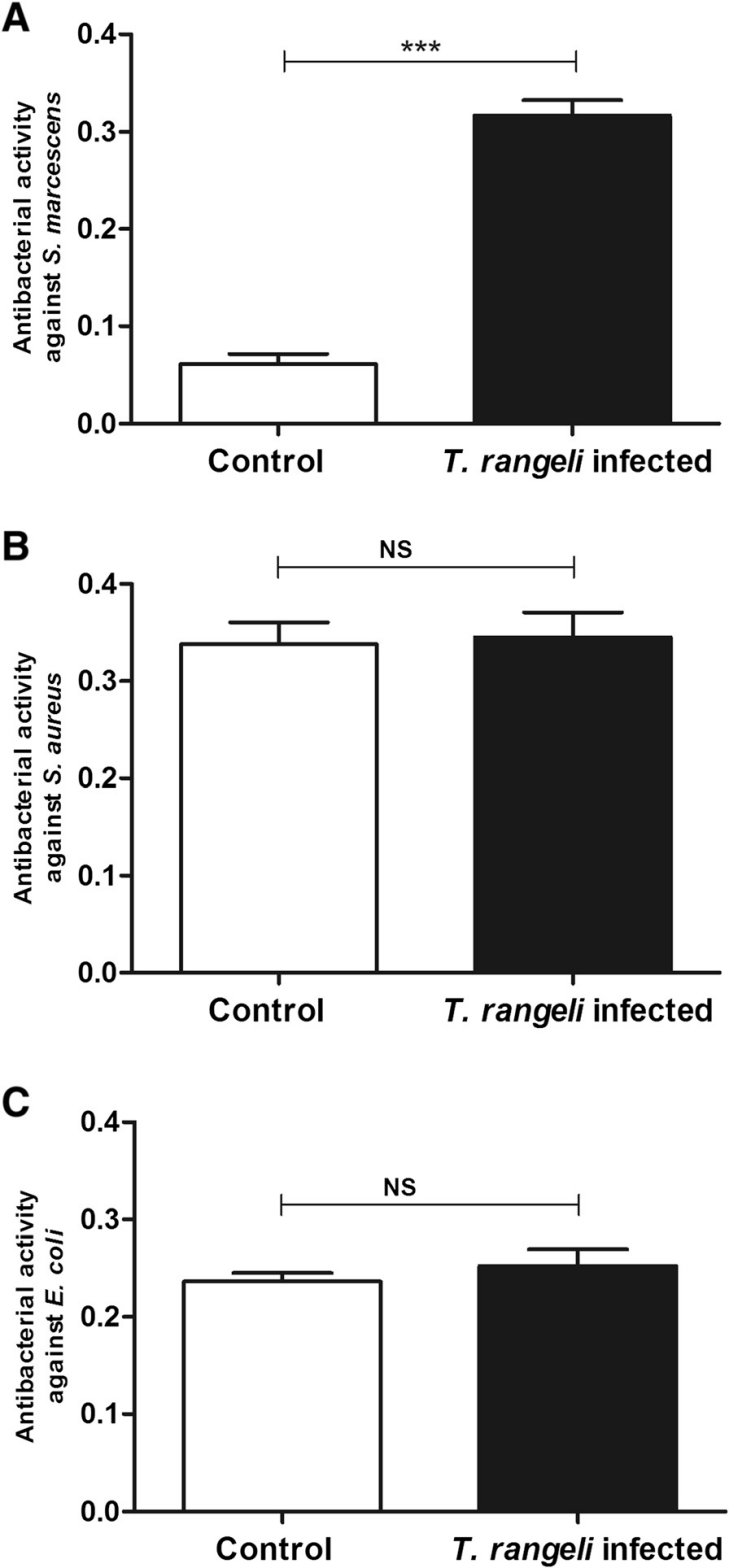
**Short-term experiment showing antibacterial activity in the midgut of** ***Rhodnius prolixus*** **infected with** ***Trypanosoma rangeli.*** The antibacterial activities of anterior midgut of *R. prolixus* 5^th^ instar nymphs 7 days after infection with *T. rangeli* were tested against **(A)** *S. marcescens* **(B)** *S. aureus* **(C)** *E. coli*. Treatments: white columns – control, uninfected group; black columns – infected group. Fifth instar nymphs were fed on inactivated blood with or without 1 × 10^6^ epimastigotes/mL. Antibacterial activity value was measured using the turbidimetric assay (TB) (OD_550_ nm) after 19 h incubation of midgut samples with different bacteria and calculated by the difference between the optical densities of the midgut samples and bacterial control. Bars represent mean ± SEM of three independent experiments. Each experiment consisted of three pools of three insects (n = 9). Means were compared using *t*-test; ***p < 0.001 and NS = not significant.

The antibacterial activity in 5^th^ instar nymphs infected with *T. rangeli* was analysed *in vitro* by incubating anterior midgut content samples collected 7 DAF with different bacterial strains. Antibacterial activity of the infected insects against *S. marcescens* was significantly higher than in control insects (p < 0.001) (Figure 3A). The activities measured against *S. aureus* and *E. coli* were similar in infected insects compared with the controls (Figure 3B, C).

#### Transcript abundance of antimicrobial peptides (AMPs)

**Figure 4:**
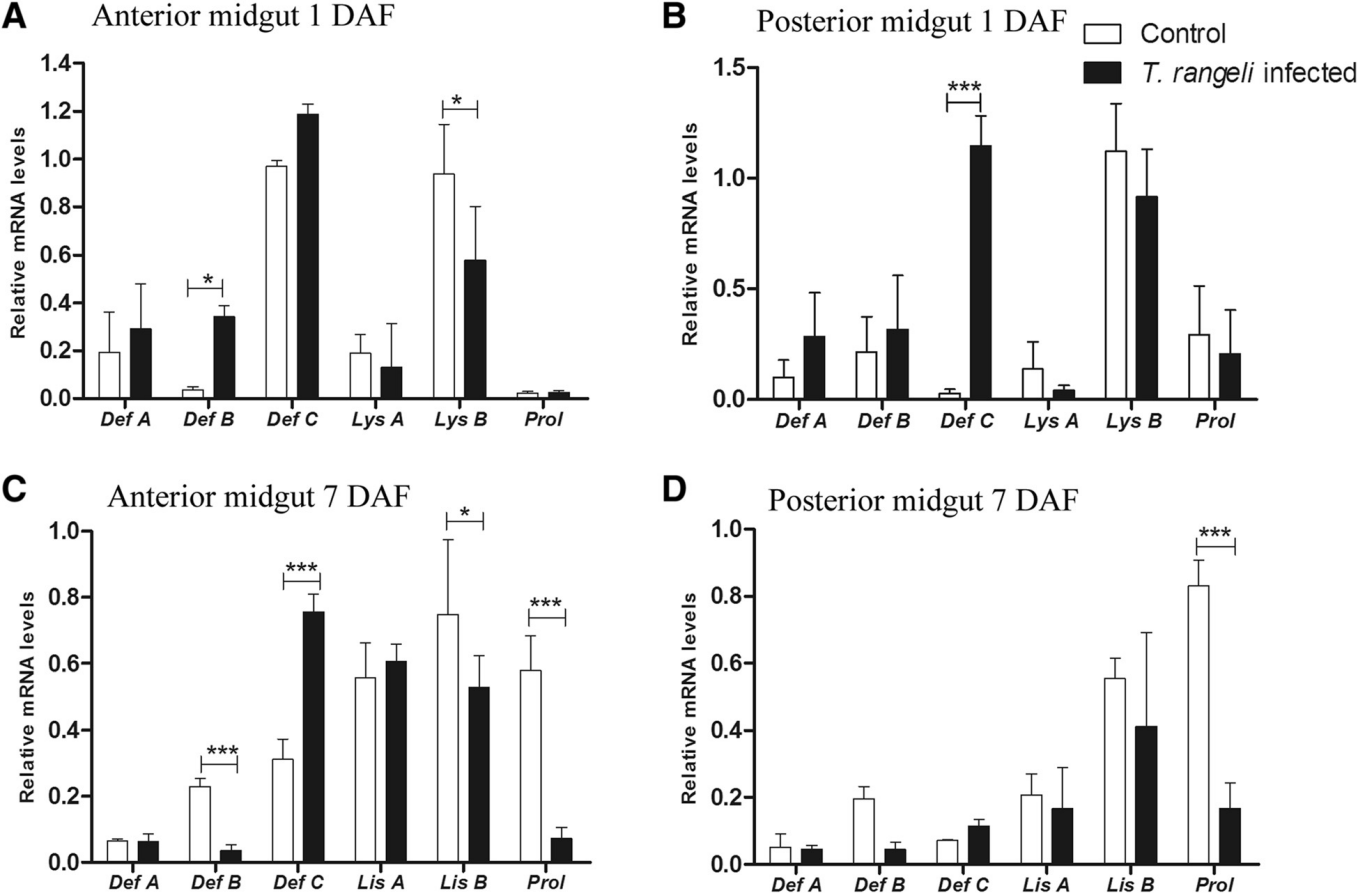
**Short-term experiment showing relative transcript abundance of antimicrobial peptides and lysozymes in** ***Rhodnius prolixus*** **midgut** ***.*** Relative AMP mRNA levels from *R. prolixus* 5^th^ instar nymphs were analysed 1 and 7 days after feeding (DAF) **A** – anterior midgut 1 DAF; **B** – posterior midgut 1 DAF; **C** – anterior midgut 7 DAF; **D** – posterior midgut 7 DAF. Treatments: white columns – control, uninfected group; black columns – infected group. Insects were fed on inactivated blood with or without 1 × 10^6^ epimastigotes/mL. Bars represent mean ± SEM of three independent experiments with a pool of 10 insects (n  = 3). Means were compared using one-way ANOVA and Student *T*-test; ***p < 0.001 and *p < 0.05.

The modification of antimicrobial activities in the anterior and posterior midguts of the 5^th^ instar nymphs infected with *T. rangeli* was analysed by the transcript abundance profiles of AMPs 1 and 7 DAF. The relative abundance of lysozyme A (*LysA*), lysozyme B (*LysB*), prolixicin (*Prol*), defensins A (*DefA*), B (*DefB*) and C (*DefC*) was also quantified (Figure 4).

In the anterior midgut at 1 DAF, the expression of *DefB* was significantly higher (p < 0.05) and *LysB* was significantly lower (p < 0.05) in comparison between infected and control insects, respectively (Figure 4A). In contrast, at 7 DAF, three AMPs (*DefB, LysB* and *Prol*) had significantly lower levels (p < 0.001; p < 0.05; p < 0.001, respectively) and only one (*DefC*) had a significantly higher level (p < 0.001) of transcripts in infected insects when compared to control (Figure 4C). However, in the posterior midgut the differences in levels of the AMPs between the infected and control insects were less striking. Compared to control the infected insects presented higher levels (p < 0.001) of *DefC* transcripts at 1 DAF and lower levels (p < 0.001) of *Prol* at 7DAF (Figure 4B and D).

#### Determination of phenoloxidase activity

**Figure 5:**
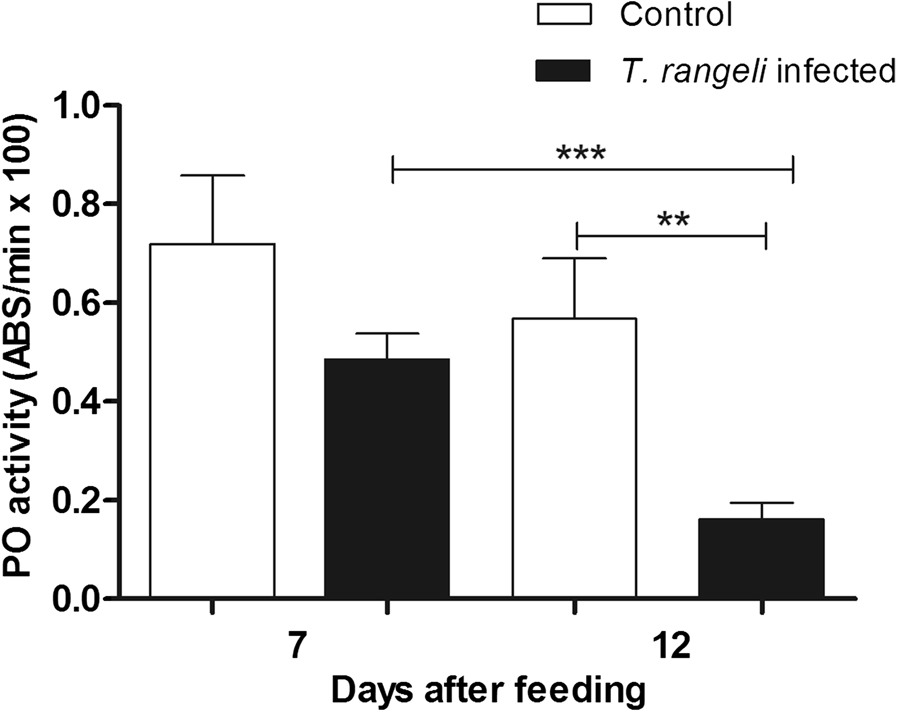
**Short-term experiment showing phenoloxidase activity in the midgut of** ***Rhodnius prolixus*** **infected with** ***Trypanosoma rangeli*** **.** PO activities were measured in the anterior midgut of *R. prolixus* 5^th^ instar nymphs at 7 and 12 days after infection with *T. rangeli*. The insects were fed on inactivated blood with or without 1 × 10^6^ epimastigotes/mL. Treatments: white columns – control, uninfected group; black columns – infected group. Bars represent mean ± SEM of three independent experiments each with five insects (n =15). Means were compared using Student *T*-test; ***p < 0.001 and **p < 0.01.

PO activities measured in the anterior midgut contents of the 5^th^ instar nymphs at 7 DAF did not show significant differences, when comparing *T. rangeli* infected and control groups. However, at 12 DAF, the PO activity was significantly lower in infected insects than in the control (p < 0.01) (Figure 5). The PO activity inhibition by *T. rangeli* infection was significantly higher at 12 DAF when compared with 7 DAF (p < 0.001) (Figure 5).

### Long term infection

#### Quantification of parasites in the digestive tract

**Figure 6:**
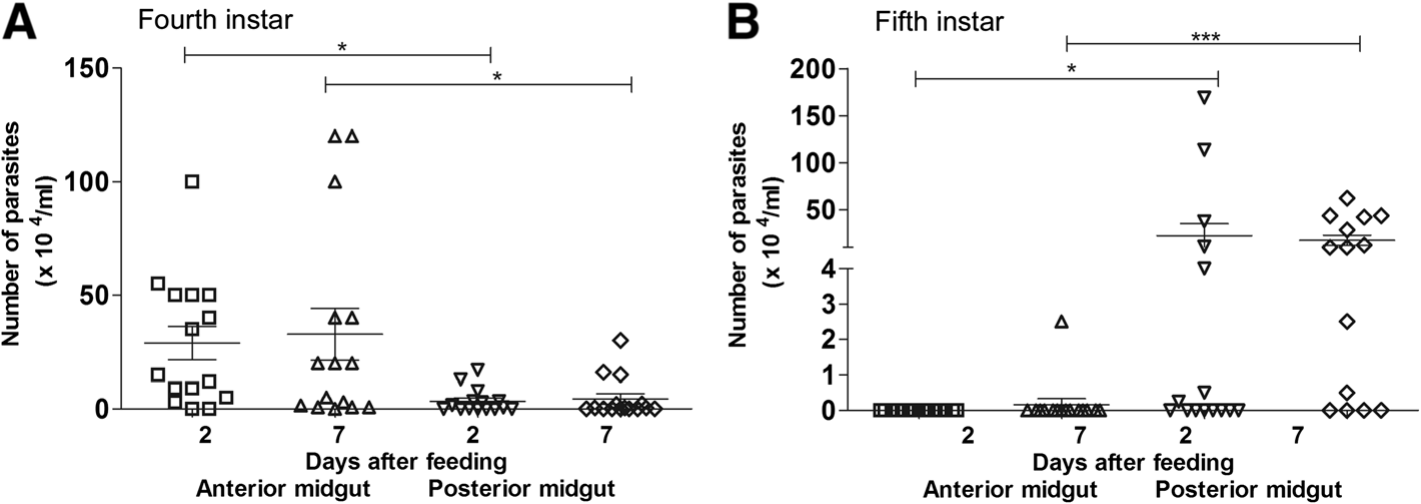
**Long-term experiment showing** ***Trypanosoma rangeli*** **numbers in the gut of** ***Rhodnius prolixus*** **.** Parasite numbers were analysed in *R. prolixus* **(A)** 4^th^ and **(B)** 5^th^ instar nymphs: anterior midgut and posterior midgut at 2 and 7 days after feeding. Fourth instar nymphs were fed on inactivated blood containing 1 × 10^6^ epimastigotes/mL. After moulting, 5^th^ instar nymphs were fed on blood without parasites. Bars represent mean ± SEM of three independent experiments each with five insects (n  = 15). Means were compared using one-way ANOVA and Kruskal-Wallis test; ***p < 0.001 and *p < 0.05.

The *T. rangeli* infection in the insects was also investigated in long-term experiments. Parasites were quantified in the digestive tract from the 4^th^ instar nymphs when infection occurred and after insects moulted to 5^th^ instar followed by a second feeding with parasite free blood. The percentages of infected insects in 4^th^ instar nymphs at 2 and 7 days after infection were 86.7% and 93.3%, respectively. In this infected group, the number of parasites encountered in the anterior midgut was significantly higher than in the posterior midgut on both days analysed (Figure 6A). The parasite numbers reached 28.9 × 10^4^/mL and 32.8 × 10^4^/mL in the anterior midgut at 2 and 7 DAF, respectively, and 3.4 × 10^4^/mL and 4.4 × 10^4^/mL in the posterior midgut at 2 and 7 DAF, respectively (Figure 6A).

The percentages of 5^th^ instar nymphs which showed *T. rangeli* infection in the digestive tract were 46.7% and 73.3% at 2 and 7 DAF, respectively, after an uninfected blood meal. In these 5^th^ instar nymphs, the results were opposite to those observed in the 4^th^ instar nymphs, in which the anterior midgut presented significantly lower numbers of parasites than the posterior midgut on both days analysed (Figure 6B). The infection level in the anterior midgut was 0 and 0.17 × 10^4^/mL at 2 and 7 DAF respectively and in the posterior midgut and rectum was 22.4 × 10^4^/mL and 17.7 × 10^4^/mL at 2 and 7 DAF, respectively (Figure 6B). These results showed that *T. rangeli* successfully colonized *R. prolixus* midgut, even after moulting and a second blood meal (Figure 6).

### Analysis of intestinal microbiota

#### Colony forming unit (CFU)

**Figure 7:**
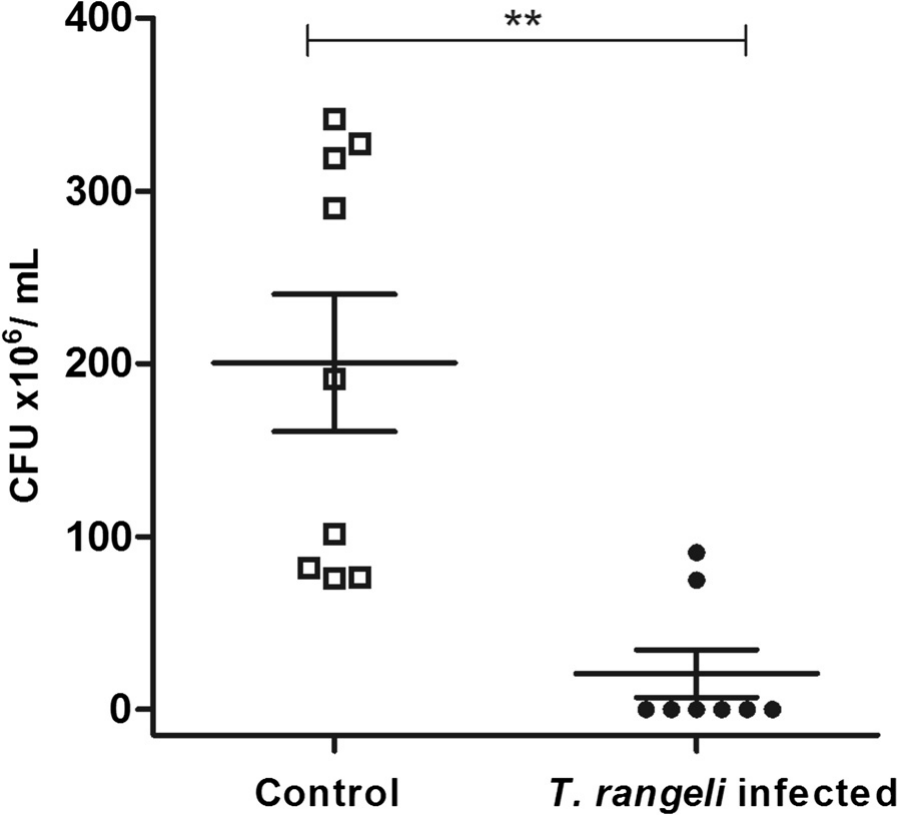
**Long-term experiment showing bacteria population in** ***Rhodnius prolixus*** **midgut infected with** ***Trypanosoma rangeli*** **.** CFU counts were made with *R. prolixus* 5^th^ instar nymphs 12 days after feeding (DAF) on blood without parasites. Previously, 4^th^ instar nymphs were fed on inactivated blood with or without 1 × 10^6^ epimastigotes/mL. Bars represent mean ± SEM of three independent experiments. Each point represents the CFU from three pools of three insects (n = 9). Means were compared using Student *T*-test or Mann–Whitney test; **p < 0.01 and *p < 0.05.

The cultivable bacterial microbiota population of *R. prolixus* 5^th^ instar nymphs, infected as 4^th^ instars with *T. rangeli* was significantly lower than control insects at 12 DAF (p < 0.01) (Figure 7).

#### Amplification and 454 sequencing of targeted 16S rRNA gene variable region

**Figure 8:**
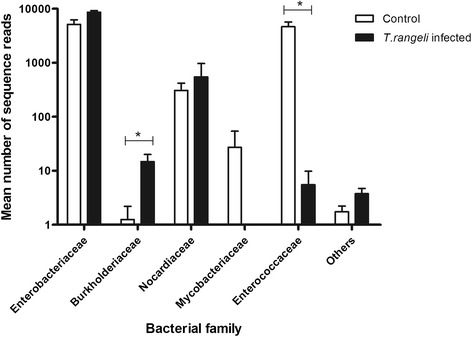
**Bacterial composition identified by 16S ribosomal pyrosequencing in** ***Rhodnius prolixus*** **midgut infected with** ***Trypanosoma rangeli*** **.** Long-term experiment showing bacterial composition at the family levels. Pyrosequencing 454 experiments of anterior midgut preparations from *R. prolixus* 5^th^ instar nymphs 7 days after feeding (DAF) on blood without parasites. Previously, 4^th^ instar nymphs were fed on inactivated blood with or without 1 × 10^6^ epimastigotes/mL of *T. rangeli*. Each bar graph presents the mean number of sequence reads assigned to a given bacterial family in four insect samples. Others: represent families with only one or two sequences (Pseudomonadaceae, Comamonadaceae, Rhodobacteraceae, Phyllobacteriaceae, Bradyrhizobiaceae, Staphylococcaceae, Bacillaceae, Nitrospiraceae, Flavobacteriaceae). Means were compared using *t*-test or Mann–Whitney test; *p < 0.05.

The bacterial microbiota in the anterior midgut was predominantly composed of Enterobacteriaceae and Enterococcaceae families, which include *Serratia* and *Enterococcus* species, respectively, as well as Nocardiaceae (Figure 8). Seven days after feeding, there was a significant decrease of Enterococcaceae in the *R. prolixus* 5^th^ instar nymphs, infected at 4^th^ instar with *T. rangeli* while there was significant increase of Burkholderiaceae in the infected 5^th^ instar nymphs (Figure 8).

### Turbidimetric antibacterial assay

**Figure 9:**
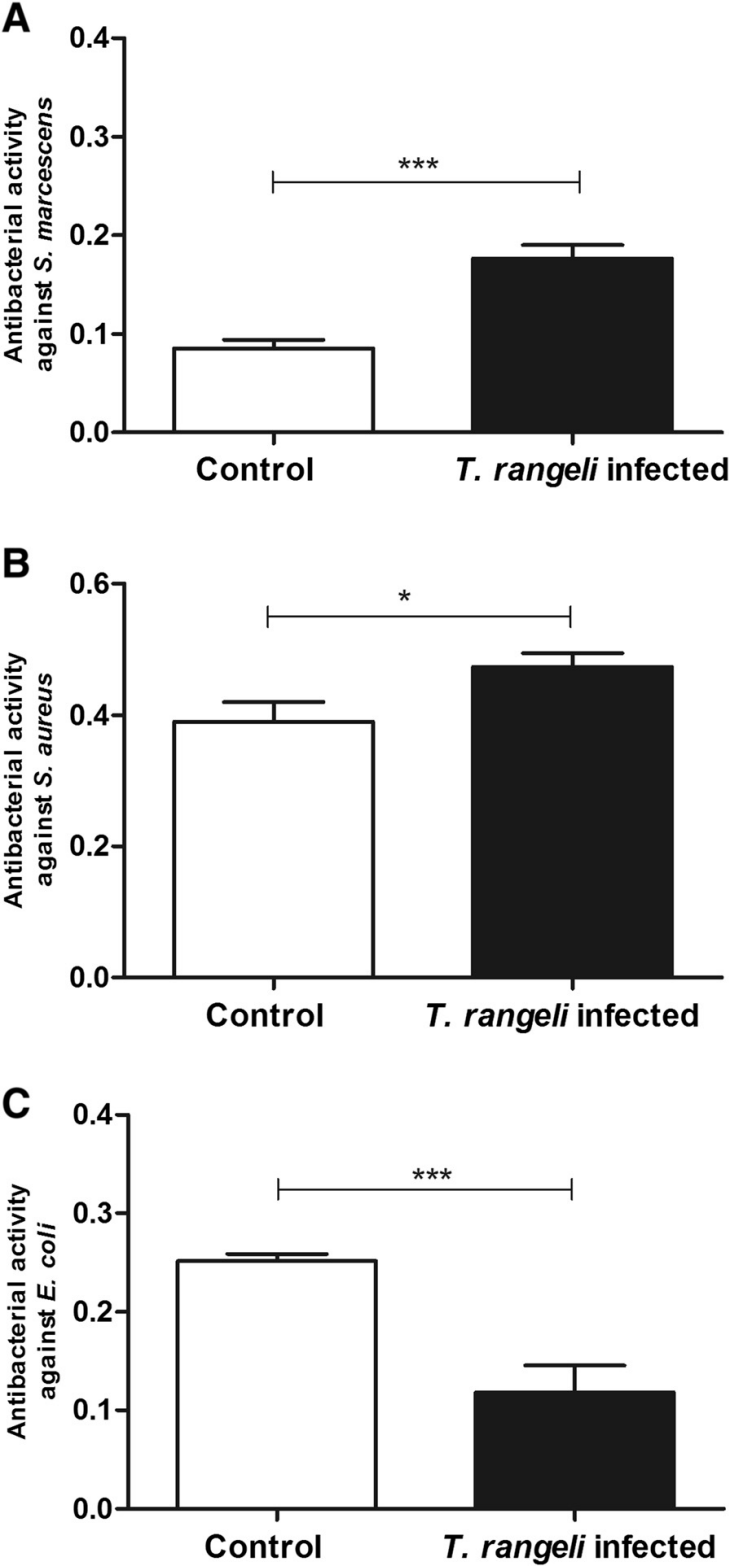
**Long-term experiment showing antibacterial activity in the midgut of** ***Rhodnius prolixus*** **infected with** ***Trypanosoma rangeli.*** Antibacterial activities of anterior midgutof *R. prolixus* 5^th^ instar nymphs 7 days after feeding (DAF) on blood without parasiteswere tested against **(A)** *S. marcescens* **(B)** *S. aureus* **(C)** *E. coli*. Treatments: white columns – control, uninfected group; black columns – infected group. Previously, 4^th^ instar nymphs were fed on inactivated blood with or without 1 × 10^6^ epimastigotes/mL. Anterior midgut contents were collected from 5^th^ instar nymphs 7 (DAF). Antibacterial activity was measured using the turbidimetric assay (TB) (OD_550_ nm) after 19 h incubation of midgut samples with different bacteria and calculated by the difference between the optical densities of the midgut samples and bacterial control. Bars represent mean ± SEM of three independent experiments. Each experiment consisted of three pools of three insects (n = 9). Means were compared using Student *T*-test; ***p < 0.001 and *p < 0.05.

The anterior midgut antibacterial activity of insects infected over the long-term was investigated. Comparing to the control group, infected insects presented significantly higher antibacterial activity against *S. marcescens* (p < 0.001) and *S. aureus* (p < 0.05) and lower activity against *E. coli* (p < 0.001) (Figure 9).

#### Transcript abundance of antimicrobial peptides (AMPs)

**Figure 10:**
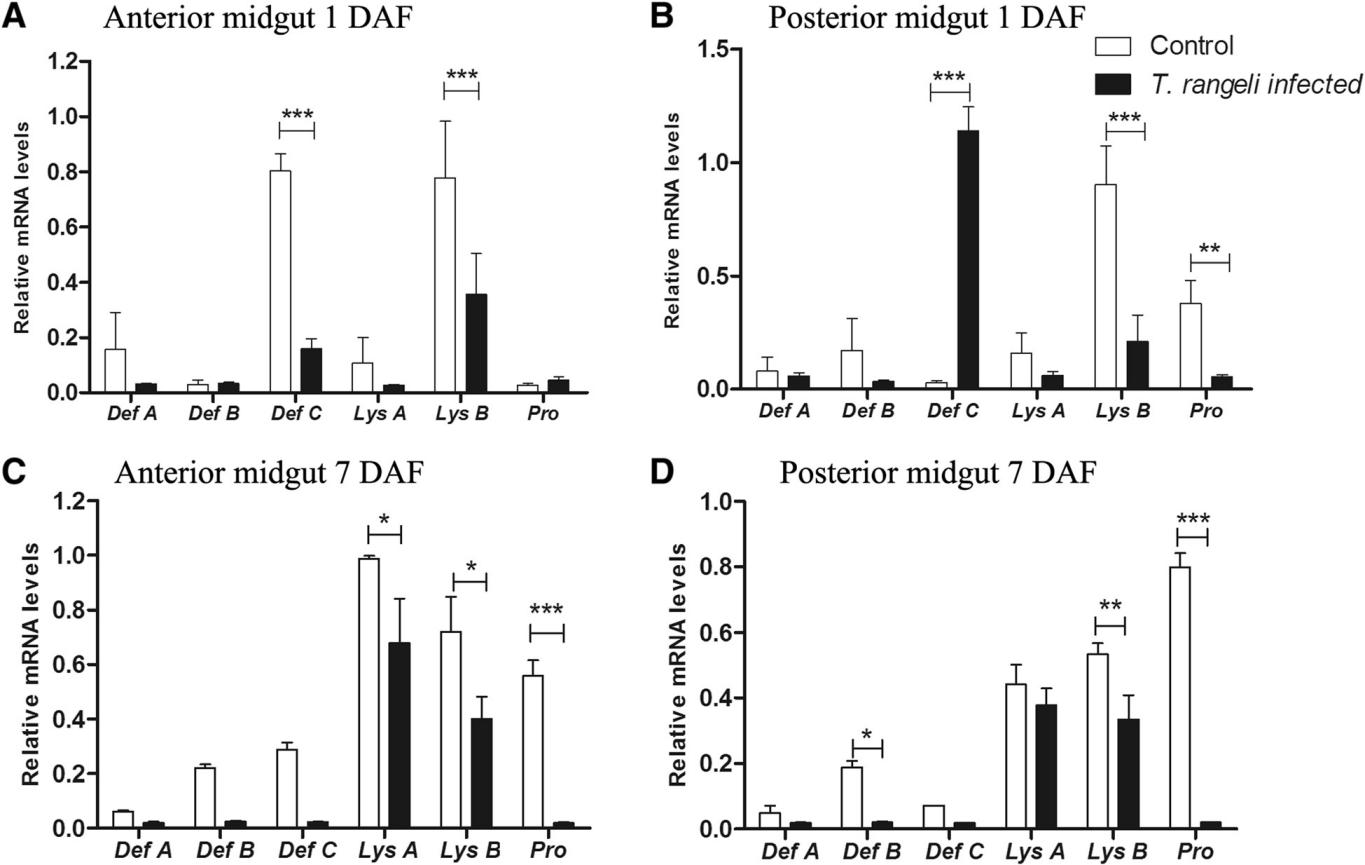
**Long-term experiment showing relative transcript abundance of antimicrobial peptides and lysozymes in** ***Rhodnius prolixus*** **midgut** ***.*** Relative AMP mRNA levels from *R. prolixus* 5^th^ instar nymphs were analysed 1 and 7 days after feeding (DAF) on blood without parasites. **A** – anterior midgut 1 DAF; **B** – posterior midgut 1 DAF; **C** – anterior midgut 7 DAF; **D** – posterior midgut 7 DAF. Previously, 4^th^ instar nymphs were fed on inactivated blood with or without 1 × 10^6^ epimastigotes/mL. Treatments: white columns – control, uninfected group; black columns – infected group. Bars represent mean ± SEM of three independent experiments with a pool of 10 insects (n  = 3). Means were compared using one-way ANOVA and Student *T*-test; ***p < 0.001, **p < 0.01 and *p < 0.05.

The relative transcript abundance of AMPs and lysozymes encoding mRNA in the 5^th^ instar *R. prolixus* nymphs that were infected with *T. rangeli* as 4^th^ instar nymphs was investigated (Figure 10). The expression of *LysB* was significantly lower in both compartments of the midgut at 1 and 7 DAF in infected insects when compared to the uninfected control group. This difference in abundance of *LysB* was more significant at 1 DAF (p < 0.001) both in the anterior and posterior midguts of the infected insects (Figure 10A and 10B). The abundance of *LysA* transcripts was significantly lower only in the anterior midgut at 7 DAF of the infected insects (p < 0.05) in comparison to the control insects (Figure 10C). Compared to the control group, *DefC* transcripts in infected insects were less abundant in the anterior midgut at 1 DAF (p < 0.001). In the posterior midgut at 7 DAF abundance of the *DefB* transcripts was lower (p < 0.01) in infected insects than in the control insects (Figure 10D). Moreover, the abundance of *Prol* was significantly lower in the anterior midgut at 7 DAF (p < 0.001) and in the posterior midgut at 1 and 7 DAF (p < 0.01) of infected insects when compared to control (Figures 10 B, C and D). Only *DefC* mRNA levels in the posterior midgut were up-regulated (57-fold, p < 0.001) at 1 DAF in infected insects when compared to the control group (Figure 10B).

#### Prophenoloxidase (PPO) activity

**Figure 11:**
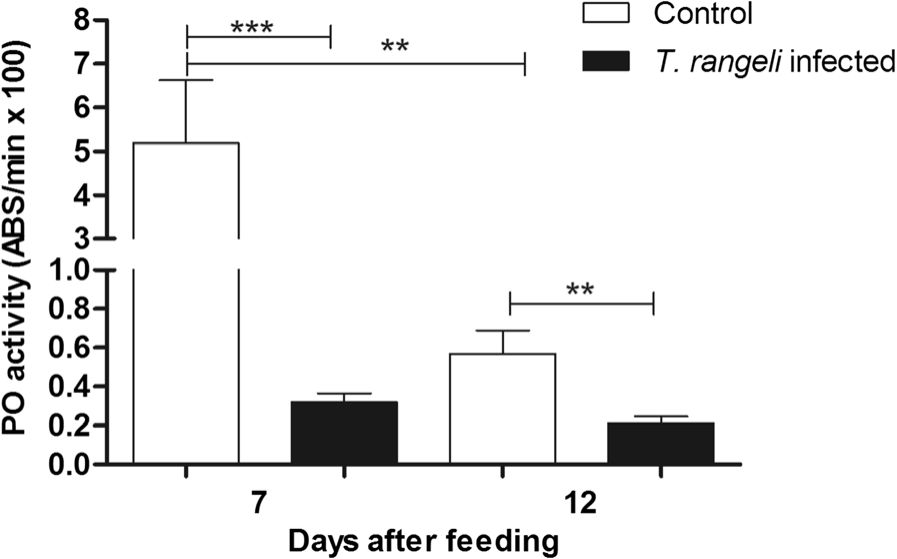
**Long-term experiment showing phenoloxidase activity in the midgut of** ***Rhodnius prolixus*** **infected with** ***Trypanosoma rangeli*** **.** PO activities were measured in the anterior midgut of *R. prolixus* 5^th^ instar nymphs at 7 and 12 days after feeding on blood without parasites. Previously, 4^th^ instar nymphs were fed on inactivated blood with or without 1 × 10^6^ epimastigotes/mL. Treatments: white columns – control, uninfected group; black columns – infected group. Bars represent mean ± SEM of three independent experiments each with five insects (n  = 15). Means were compared using Student *T*-test; ***p < 0.001 and **p < 0.01.

The anterior midgut of 5^th^ instar nymphs, previously infected with *T. rangeli* at 4^th^ instar, were investigated. The PO activities of infected insects were significantly lower than the control insects at 7 and 12 DAF (p < 0.001 and p < 0.01, respectively) (Figure 11). Moreover, the PO activity in the control insects was lower at 12 DAF when compared to 7 DAF (p < 0.01) (Figure 11).

## Discussion

Experiments in which *R. prolixus* were infected with *T. rangeli* H14 or Choachi strains have demonstrated that the modulation of the insect’s immune responses and subsequent establishment of the infection in the digestive tract depends on the strain of the parasite [25,40-44]. It is also known that gut microbiota can be correlated to the success of the parasite infection in diverse invertebrate hosts [17,45-48]. Therefore, we infected *R. prolixus* with the *T. rangeli* Macias strain and investigated the modulation of the immune system and bacteria population of the insect’s digestive tract. Our results demonstrated that the percentage of insects with intestinal parasites varied within days after infection and midgut compartments examined. In the short-term infection, *T. rangeli* predominantly colonized the anterior midgut and the number of parasites decreased over time.

In the long-term experiments, the parasites were found preferentially in the anterior midgut of the 4^th^ instar nymphs; however after moulting and receiving a parasite-free blood meal, *T. rangeli* was predominantly found in the posterior midgut of the 5^th^ instar nymphs. In terms of *T. cruzi* development in triatomines*,* the parasites migrate to the posterior midgut and rectum within few weeks after infection [49-52]. Depending on the nutritional conditions of the insect, the anterior midgut contains different microbiota compositions and cytotoxic components (nitrogen and oxygen reactive species, AMPs and haemolysins) that may create a hostile environment for the parasites [20,36,51,53-56].

It is known that the intestinal microbiota modulates the host immune responses and can interfere in parasite infection [12,57,58]. Moreover, the bacteria density can be regulated depending on the parasite genotype infecting the insect host as observed with *T. cruzi* infection in *R. prolixus* [17]. The present study showed lower cultivable bacterial CFU numbers in the digestive tract of *R. prolixus* infected with *T. rangeli* than in control insects (short and long-term). Additionally, bacterial microbiota analysis by pyrosequencing revealed a decrease of Enterococcaceae and Mycobacteriaceae while Burkholderiaceae increased in sequence numbers in infected insects. In other insect vectors, such as *Glossina* and *Anopheles,* bacteria from these families have also been observed and they have varied with parasite infection as well [59,60]. *Rhodococcus rhodnii* and *S. marcenscens* that have been frequently observed colonizing triatomines [12,61,62] and which belong to the families Nocardiaceae and Enterobacteriaceae, respectively, were not altered after *T. rangeli* Macias strain infection in *R. prolixus*. However, *R. rhodnii* population decreased in a study using the *T. rangeli* Choachi strain infecting *R. prolixus* [63]. Additionally, *in vitro* studies have already shown that *S. marcescens* possess cytotoxicity against some *T. cruzi* and *T. rangeli* strains [12,13,55,64,65] besides its antibiotic activity [66]. These findings indicate that bacterial communities can be modulated differently depending on the *T. rangeli* strain.

We analysed the antibacterial activity in the midgut of the *T. rangeli* infected insects and showed that this activity was related to the decrease in the bacteria population of the insect’s digestive tract. The high antibacterial activity observed against *S. marcescens in vitro* can be one reason for the low number of cultivable bacteria detected in the CFU analysis. In addition, the high antimicrobial activity against *S. aureus* might reflect the decrease of *Enterococcus* in the midgut of infected samples analysed by pyrosequencing.

The production of AMPs in the insect gut has been demonstrated to be vital to maintain insect homeostasis of the intestinal microbiota which provide essential nutrients, promote digestion and control pathogenic microorganisms by modulating the immune responses [55,67-69]. In *Drosophila* the activation of signalling pathways of the immunity depends on the type of predominant microorganisms in the digestive tract [70-72].

An important immune response in the midgut lumen of insect vectors to control natural microbiota growth and pathogens is the production of AMPs [55,69]. AMPs are effectors molecules of the humoral immune system of insects that control microorganisms by disrupting cell membranes [73-75]. Analysis of the relative expression of mRNAs encoding lysozymes and AMPs in *T. rangeli* infected insects showed a different pattern in short and long-term infections. However, in general there was a suppression of most AMP genes. For example, *LysB* and *LysA* down-regulation was observed in the anterior and posterior midgut compartments. A previous work suggested that *LysA* is mainly expressed in the midgut with a digestive function while *LysB* is expressed in the fat body with an immune role [38]. Nevertheless, *S. aureus* oral infection in *R. prolixus* increased *LysA* mRNA levels in the midgut [36]. Combining these results with the suppression of *LysA* by *T. rangeli* infection observed herein, we suggest its involvement in the immune response.

Regarding prolixicin, a previous work showed that this peptide presented antimicrobial activities against Gram-negative and Gram-positive bacteria, but no toxicity against *T. cruzi* was detected [19]. In the present work, *Prol* was down-regulated in both midgut compartments, in both the short and long-term infections with *T. rangeli*. Although cytotoxicity of prolixicin against *T. rangeli* has not been described in the literature, the present results suggest that the modulation of *Prol* expression by *T. rangeli* could be one possible mechanism that, indirectly benefits the parasite’s development in *R. prolixus*.

Another group of AMPs extensively studied in insects are the defensins. These peptides are known to act mainly on Gram-positive bacteria, but also show some activity against Gram-negative bacteria [76,77] and some protozoans such as *Plasmodium* and *Trypanosoma* [78-81]. Considering the short-term infection and the parasite population dynamics in the insect’s midgut, a rapid increase of *DefB* levels and its subsequent down-regulation in the anterior midgut suggests a possible role of the respective peptides in the control of microorganism density in this compartment. The role of defensins in the control of trypanosomatid infections in the vector has been suggested previously [80,82]. On the other hand, the increase of *DefC* in both midgut compartments represents an immune modulation caused by *T. rangeli* that could represent a strategy to facilitate the establishment of *T. rangeli* in the gut of *R. prolixus*. Combined, these results suggest that an increase of antimicrobial activities and a decrease of CFU numbers detected in the anterior midgut in short term infection might be a result of the increased *DefB* and *DefC* levels observed. Long-term infection resulted in a massive up-regulation of *DefC* in the posterior midgut, which can explain the decrease of bacteria population encountered and the parasite’s preference to develop in this midgut compartment. These results indicate that the parasite infection can modulate the insect’s immune system, which consequently can influence the microbiota population in the insect’s digestive tract.

Another important biological event in the *T. rangeli* cycle, in its invertebrate host, is its ability to modulate the PPO system in the triatomine haemolymph [83-85].The presence of *T. rangeli* also reduced the level of PPO activation *in vitro* [86] and *in vivo* in *R. prolixus* haemolymph [22,84]. The present study is the first to demonstrate that the PO activity in the *R. prolixus* midgut was also inhibited after oral infection with *T. rangeli.* The PO activity in the midgut seems to be differently regulated accordingly to the trypanosomatid species. While *T. rangeli* has the ability to inhibit the insect PPO system, *T. cruzi* infection induces an increase in this immune response [17]. Other immune modulated factors such as reactive oxygen and nitrogen species may be involved in the development of the parasite in insect’s midgut [17,87-89].

## Conclusion

Parasite-microbiota competition for nutrients can change the bacteria composition in the *R. prolixus* midgut and subsequently modulate the insect’s immune system. A direct modulation of the immune system by the parasite can also affect the microbiota population. The strategy of certain trypanosome species for successful infection of the invertebrate host is a complex interplay and depends on a tripartite interaction between parasite, insect immune system and bacteria [46,59,60,90,91]. These interactions are an important field for research, opening up new insights into the understanding of parasite-vector relationships [92]. A better understanding of the role of bacterial species composing the gut microbiota on host immunity against pathogens can lead to the development of new strategies to control vector-borne diseases.
